# A review of the reporting and handling of missing data in cohort studies with repeated assessment of exposure measures

**DOI:** 10.1186/1471-2288-12-96

**Published:** 2012-07-11

**Authors:** Amalia Karahalios, Laura Baglietto, John B Carlin, Dallas R English, Julie A Simpson

**Affiliations:** 1 Cancer Epidemiology Centre, Cancer Council Victoria, Carlton, VIC, Australia; 2 Centre for Molecular, Environmental, Genetic, and Analytic Epidemiology, School of Population Health, The University of Melbourne, Parkville, VIC, Australia; 3 Clinical Epidemiology and Biostatistics Unit, Murdoch Children’s Research Institute, Parkville, VIC, Australia

**Keywords:** Longitudinal cohort studies, Missing exposure data, Repeated exposure measurement, Missing data methods, Reporting

## Abstract

**Background:**

Retaining participants in cohort studies with multiple follow-up waves is difficult. Commonly, researchers are faced with the problem of missing data, which may introduce biased results as well as a loss of statistical power and precision. The STROBE guidelines von Elm et al. (Lancet, 370:1453-1457, 2007); Vandenbroucke et al. (PLoS Med, 4:e297, 2007) and the guidelines proposed by Sterne et al. (BMJ, 338:b2393, 2009) recommend that cohort studies report on the amount of missing data, the reasons for non-participation and non-response, and the method used to handle missing data in the analyses. We have conducted a review of publications from cohort studies in order to document the reporting of missing data for exposure measures and to describe the statistical methods used to account for the missing data.

**Methods:**

A systematic search of English language papers published from January 2000 to December 2009 was carried out in PubMed. Prospective cohort studies with a sample size greater than 1,000 that analysed data using repeated measures of exposure were included.

**Results:**

Among the 82 papers meeting the inclusion criteria, only 35 (43%) reported the amount of missing data according to the suggested guidelines. Sixty-eight papers (83%) described how they dealt with missing data in the analysis. Most of the papers excluded participants with missing data and performed a complete-case analysis (n = 54, 66%). Other papers used more sophisticated methods including multiple imputation (n = 5) or fully Bayesian modeling (n = 1). Methods known to produce biased results were also used, for example, Last Observation Carried Forward (n = 7), the missing indicator method (n = 1), and mean value substitution (n = 3). For the remaining 14 papers, the method used to handle missing data in the analysis was not stated.

**Conclusions:**

This review highlights the inconsistent reporting of missing data in cohort studies and the continuing use of inappropriate methods to handle missing data in the analysis. Epidemiological journals should invoke the STROBE guidelines as a framework for authors so that the amount of missing data and how this was accounted for in the analysis is transparent in the reporting of cohort studies.

## Background

A growing number of cohort studies are establishing protocols to re-contact participants at various times during follow-up. These waves of data collection provide researchers with the opportunity to obtain information regarding changes in the participants’ exposure and outcome measures. Incorporating the repeated measures of the exposure in the epidemiological analysis is especially important if the current exposure (or change in exposure) is thought to be more predictive of the outcome than the participants’ baseline measurement [1] or the researcher is interested in assessing the effect of a cumulative exposure [2]. The time frames for these follow-up waves of data collection can vary from one to two years up to 20 to 30 years or even longer post-baseline. Repeated ascertainment of exposure and outcome measures over time can lead to missing data for reasons such as participants not being traceable, too sick to participate, withdrawing from the study, refusing to respond to certain questions or death [3,4]. In this paper we focus on missing data in exposure measures that are made repeatedly in a cohort study because studies of this type (in which the outcome is often a single episode of disease or death obtained from a registry and therefore, known for all participants) are common and increasingly important in chronic disease epidemiology. Further research is needed on the consequences of and best methods for handling missing data in such study designs, but simulation and case studies have shown that missing covariate data can lead to biased results and there may be gains in precision of estimation of effects if multiple imputation is used to handle missing covariate data [5-7].

If participants with missing data and complete data differ with respect to exposure and outcome, estimates of association based on fully observed cases (known as a complete-case analysis) might be biased. Further, the estimates from these analyses will have less precision than an analysis of all participants in the absence of missing data. As well as complete-case analysis, there are other methods available for dealing with missing data in the statistical analysis [8,9]. These include *ad hoc* methods such as Last Observation Carried Forward and the missing indicator method, and more advanced approaches such as multiple imputation and likelihood-based formulations.

The STROBE guidelines for reporting of observational studies, published in 2007, state that the method for handling missing data should be addressed and furthermore, that the number of individuals used for analysis at each stage of the study should be reported accompanied by reasons for non-participation or non-response [10,11]. The guidelines published by Sterne et al. [12], an extension to the STROBE guidelines, provide general recommendations for the reporting of missing data in any study affected by missing data and specific recommendations for reporting the details of multiple imputation.

In this paper we: 1) give a brief review of the statistical methods that have been proposed for handling missing data and when they may be appropriate; 2) review how missing exposure data has been reported in large cohort studies with one or more waves of follow-up, where the repeated waves of exposures were incorporated in the statistical analyses; and 3) report how the same studies dealt with missing data in the statistical analyses.

## Methods

### Statistical methods for handling missing data

*Complete-case analysis* only includes in the analysis participants with complete data on all waves of data collection, thereby potentially reducing the precision of the estimates of the exposure-outcome associations [2]. The advantage of using complete-case analysis is that it is easily implemented, with most software packages using this method as the default. The estimates of the associations of interest may be biased if the participants with missing data are not similar to those with complete data. To be valid, complete-case analyses must assume that participants with missing data can be thought of as a random sample of those that were intended to be observed (commonly referred to in the missing data nomenclature as missing completely at random (MCAR) [13]), or at least that the likelihood of exposure being missing is independent of the outcome given the exposures [5].

There are three commonly used *ad hoc* approaches for handling missing data, all of which can lead to bias [3,12,14]. The *Last Observation Carried Forward (LOCF)* method replaces the missing value in a wave of data collection with the non-missing value from the previous completed wave for the same individual. The assumption behind this approach is that the exposure status of the individual has not changed over time. The *mean value substitution* method replaces the missing value with the average value calculated over all the values available from the other waves of data collection for the same individual. Both LOCF and mean value substitution falsely increase the stated precision of the estimates by failing to account for the uncertainty due to the missing data and generally give biased results, even when the data are MCAR [7,15]. The *Missing Indicator Method* is applied to categorical exposures and includes an extra category of the exposure variable for those individuals with missing data. Indicator variables are created for the analysis, including an indicator for the missing data category [16]. This method is simple to implement, but also produces biased results in many settings, even when the data are MCAR [6,12].

*Multiple Imputation (MI)* begins by imputing values for the missing data multiple times by sampling from an imputation model (using either chained equations [17,18] or a multivariate normal model [19]). The imputation model should contain the variables that are to be included in the statistical model used for the epidemiological analysis, as well as auxiliary variables that may contain information about the missing data, and a “proper” imputation procedure incorporates appropriate variability in the imputed values. The imputation process creates multiple ‘completed’ versions of the datasets. These ‘completed datasets’ are analysed using the appropriate statistical model for the epidemiological analysis and the estimates obtained from each dataset are averaged to produce one overall MI estimate. The standard error for this overall MI estimate is derived using Rubin’s rules, which account for variability between-and within- the estimates obtained from the separate analyses of the ‘completed datasets’ [3,13]. By accounting for the variability between the completed (imputed) datasets, MI produces a valid estimate of the precision of the final MI estimate. When the imputation is performed using standard methods that are now available in many packages, with appropriate model specifications to reflect the structure of the data, the resulting MI estimate will be valid (unbiased parameter estimates with nominal confidence interval coverage) if the missing data are ‘Missing At Random’ (MAR) [5]. MAR describes a situation where the probability of being missing for a particular variable (e.g. waist circumference) can be explained by other observed variables in the dataset, but is (conditionally) independent of the variable itself (that is, waist circumference) [13]. On the other hand, MI may produce biased estimates if the data are ‘Missing Not At Random’ (MNAR), which occurs when the study participants with missing data differ from the study participants with complete data in a manner that cannot be explained by the observed data in the study [13].

MI is now implemented in many major statistical packages (including Stata [20] and SAS [21]) making it an easily accessible method. However, it can be a time-intensive process to impute multiple datasets, analyse the ‘completed datasets’ and combine the results; and the imputation model can be complex since it must contain the exposure and outcome variables included in the analysis model, auxiliary variables and any interactions that will be included in the final analysis model [22,23]. Sterne et al. [12] have described a number of pitfalls that can be encountered in the imputation procedure that might lead to biased results for the epidemiological analysis of interest.

Missing data can also be handled with the following more sophisticated methods: maximum likelihood-based formulations, fully Bayesian models and weighting methods. Likelihood-based methods use all of the available information (i.e. information from participants with both complete and incomplete data) to simultaneously estimate both the missing data model and the data analysis model, eliminating the need to handle the missing data directly [3,8,24,25], although in many cases the MAR assumption is also invoked to enable the missing data model to be ignored. Bayesian models also rely on a fully specified model that incorporates both the missingness process and the associations of interest [12,15,26]. Weighting methods apply weights that correspond to the inverse probability of a data observation being observed, to the observed data to account for the missing data [22,25]. These methods may improve the precision of the estimates compared with complete-case analysis. However, they are also dependent on assumptions about the missingness mechanism and in some cases on specifying the correct missingness model. In general, these methods require tailored programming which can be time consuming and requires specialist expertise [15].

### Criteria for considering studies in this review

For this review we selected prospective cohort studies that analysed exposure data collected after initial recruitment during the follow-up period (i.e. studies looking at a change in exposure or at a time varying covariate). We restricted our review to cohort studies with more than 1,000 participants, as we thought it was more likely for there to be more missing data in follow-up measurements of exposures in large cohort studies (typically population based studies) compared to small cohorts (often based on a specific clinical population). For cohort studies reported in multiple papers, we included only the most recent original research article. Studies that only used data collected at baseline or at one of the follow-up waves in the analysis, and studies that newly recruited participants at one of the waves after baseline were excluded. We did not place any restrictions on the types of exposures or outcomes studied or the type of statistical analysis performed.

### Search strategy

PubMed was searched for English language papers published between January 2000 and December 2009. We chose January 2000 as a starting date because the first widely available statistical software package for implementing MI, the NORM package [27], was developed in 1997 and updated in 1999. Search terms included: “Cohort Studies”[MeSh] AND (“attrition” OR “drop out” OR “longitudinal change” OR “missing data” OR “multiple exposure” OR “multiple follow-up” OR “multiple waves” OR “repeated exposure” OR “repeated follow-up” OR “repeated waves” OR “repeated measures” OR “time dependent covariates” OR “time dependent” OR “time varying covariate” OR “cumulative average”).

We carried out a further search of cohort studies listed in the web appendix of the paper by Lewington et al. [28], to ensure that any known large cohort studies were not missed in the original PubMed search. These cohort studies were established in the 1970s and 1980s, allowing them time to measure repeated waves of exposure on their participants and to publish these results during our study period (i.e. between 2000 and 2009).

### Methods of the review

AK reviewed all articles; any uncertainties regarding the statistical method used to handle the missing data were resolved by discussion with JAS, and AK extracted the data. Additional tables and methods sections from journal websites were checked if referred to in the article.

Our aim was to assess the reporting of missing data and the methods used to handle the missing data according to the recommendations given by the STROBE guidelines [10,11] and Sterne et al. [12]. The information extracted is summarised in Tables 1 and 2 and Additional file 1: Table S1.

**Table 1:** Summary of cohort studies included in the review

Characteristics	Number of studies (N = 82)	(%)
**Publication year**		
2000	2	(2)
2001	4	(5)
2002	4	(5)
2003	5	(6)
2004	6	(7)
2005	8	(10)
2006	11	(13)
2007	13	(16)
2008	13	(16)
2009	16	(20)
**Number of participants recruited at baseline**		
Not stated in paper	4	(5)
1,000–2,000	15	(18)
2,000–3,000	13	(16)
3,000–5,000	14	(17)
5,000–10,000	13	(16)
10,000–20,000	14	(17)
20,000 +	9	(11)
**Date of baseline recruitment (decade recruitment started)**		
Not stated in paper	5	(6)
Before 1970	9	(11)
1970–1979	9	(11)
1980–1989	25	(30)
1990–1999	30	(37)
2000–2009	4	(5)
**Number of follow-up waves used in the analysis**		
Number not stated	12	(15)
Mean or range given	7	(9)
1	10	(12)
2	9	(11)
3	18	(22)
4	12	(15)
≥5	14	(17)
**Statistical methods for epidemiological analysis†**		
Cox proportional hazards regression^	37‡	
Time-varying covariates	35	
Time-invariant covariates	3	
Generalised Estimating Equations	12*	
Linear regression	3	
Logistic regression	10	
Generalised linear mixed-effects modelling	16	
Linear regression	13	
Logistic regression	3	
Standard linear regression	3	
Standard logistic regression	9	
Other methods	6	

† The total number of papers is 83 because one paper used two analysis models: a Cox proportional hazards model for a time to event outcome and a linear mixed effects model for a numerical outcome.

^ Note one paper used a parametric survival model.

‡ One paper incorporated their repeated measures of exposures using both a time varying covariate and time-invariant covariates.

*One paper had a numerical and binary outcome.

**Table 2:** Missing data features reported by the studies

**Features reported**	**Number of papers (N = 82) (%)**
**Was the amount of missing data reported?**	
Yes	66 (80%)
Missing data reported for each follow-up wave used in the analysis	35
A general statement was made about the amount of missing data or the amount of completed follow-up (how many participants attended at least one wave or only the final follow-up wave)	22
Indicated number that completed all waves of follow-up (i.e. number included in final sample)	6
Indicated amount missing for certain (key) variables	3
No	16 (20%)
**Assessed differences between individuals with complete data and those with incomplete data?**	
Yes	26 (32%)
Provided a table comparing distributions of key exposures and outcome variables for those with missing and non-missing information	6
Table not provided but some summary statistics included in text	4
General comment provided (did not include a table or summary statistics or included p-values only)	16
No	56 (69%)
**Reasons were given for the missing data**	13 (16%)
**Statistical method for handling missing data**†	
Method not stated	14 (16%)
Complete-case analysis assumed	9 (11%)
Complete-case analysis	54 (66%)
Weighted	1
Unweighted	53
Exclude participants with missing data at any repeated waves of exposure	38
Exclude participant data record for waves of data collection with missing exposure data††	15
Missing Indicator Method	1 (1%)
Mean value substitution	3 (4%)
Last Observation Carried Forward	7 (9%)
Multiple Imputation	5 (6%)
Details provided for the multiple imputation:	
Indicated how many imputations were performed	4
Indicated which variables were included in the imputation model	2
Compared results from multiple imputation with complete case analysis	3
Performed a sensitivity analysis under different assumptions for missing data	4
Fully Bayesian Model	1 (1%)

† Three papers used more than one method to handle their missing exposure data.

†† These studies assessed both exposure and outcome measures repeatedly over the waves of data collection. The data were analysed using either Generalised Estimating Equations (GEEs) or mixed-effects models to account for the correlated outcome data within an individual and excluded participant data records for waves where the exposure data were missing.

## Results

### Study selection

**Figure 1:**
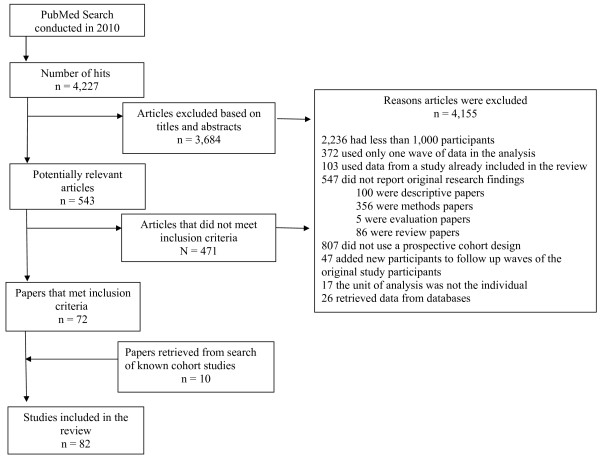
Search results.

We identified 4,277 articles via the keyword search. A total of 3,684 articles were excluded based on their title and abstract, leaving 543 articles for further evaluation. Of these, 471 articles were excluded and 72 articles were found to be appropriate for the review. A further ten studies were identified from the reference list of Lewington et al. [28] (Figure1), giving 82 studies included in this review. The reasons for excluding studies are outlined in Figure1, the most common reasons were sample size of less than 1,000 participants (54%), study design was not a prospective cohort (19%), and did not report original research findings (13%).

### Characteristics of included studies

The characteristics of the 82 studies included are summarised in Table1 and further details can be found in the additional table (see Additional file 1: Table S 1). The studies included ranged from smaller studies that recruited 1,000 to 2,000 participants at baseline to larger studies with more than 20,000 participants, and the number published annually increased steadily from two papers in 2000 to 16 papers in 2009. The majority of studies recruited their participants in the decades 1980 to 1989 (n = 25), and 1990 to 1999 (n = 30). Cox proportional hazards regression was the most common statistical method used for the epidemiological analysis (n = 37) to analyse the repeated measures of exposure, with 35 of these papers incorporating the repeated exposure(s) as a time varying covariate and the remaining two papers including a single measure of the covariate derived from repeated assessments. Generalised Estimating Equations with a logistic (n = 10) or linear regression (n = 3) and generalised linear mixed-effects models (logistic regression (n = 3) and linear regression (n = 13)) were the next most common epidemiological analyses used.

### Missing covariate data at follow-up

The methods used by the selected papers for handling missing data are summarised in Table2. Sixty-six papers (80%) commented on the amount of missing data at follow-up. Of these, only 35 papers provided information about the proportion of participants lost to follow-up at each wave. The remaining 31 papers provided incomplete details about the amount of missing data at each wave: 22 papers made a general comment about the amount of missing data; six papers reported the amount of missing data for the final wave but gave no detail regarding the number of participants available at previous waves of data collection (including baseline); and three papers only reported the amount of missing data for a few of the variables.

Of the 29 papers published after 2007, nine papers did not state the proportion of missing data at each follow up wave, three papers provided a comment as to why the data were missing and eight papers compared the baseline covariates for those with and without missing covariate data at the repeated waves of follow up.

Among those papers that provided information on missing data, the proportion of covariate data missing at any follow-up wave ranged from 2% to 65%. Twenty-six papers (32%) compared the key variables of interest for those who did and did not have data from post-baseline waves, but only six of these presented the results in detail while the rest commented briefly in the text on whether or not there was a difference.

### Methods used to deal with missing data at follow-up

The most common methods used to deal with missing data were complete-case analysis (n = 54), LOCF (n = 7) and MI (n = 5). Of the 54 papers that used complete-case analysis: 38 excluded participants who were missing exposure data at any of the waves of data collection from the analysis; one paper also excluded participants with any missing exposure data but used a weighted analysis to deal with the missing data; and the remaining 15 papers, where both the exposure and outcome measures were assessed repeatedly at each wave of data collection, excluded participant data records for waves where the exposure data were missing. Fourteen papers did not state the method used to deal with the missing data, although nine of these papers performed a Cox regression model using SAS [21] or Stata [20] and we therefore assumed that they used a complete-case analysis (Table2). Both papers published in 2000 used complete-case analysis. From 2001 to 2009, the proportion of papers using complete-case analysis ranged from 25% to 65%. Methods known to produce biased results (i.e. LOCF, the missing indicator method and mean value substitution) continue to be used, with four papers using these methods in 2009.

Of the five papers that used MI [29-33], two papers [29,30] compared the characteristics of the participants with and without missing data. For the MI, three of the five papers [30,31,33] provided details of the imputation process including the number of imputations performed and the variables included in the imputation model, and compared the results from the MI analysis to results from complete-case analysis. The other two papers [29,32] provided details about the number of imputations performed but did not describe the variables included in their imputation model and did not compare the MI results to the complete-case analysis.

## Discussion

We identified 82 cohort studies of 1,000 or more participants that were published from 2000 to 2009 and which analysed exposure data collected from repeated follow-up waves. The reporting of missing data in these studies was found to be inconsistent and generally did not follow the recommendations set out by the STROBE guidelines [10,11] or the guidelines set out by Sterne et al. [12]. The STROBE guidelines recommend that authors report the number of participants who take part in each wave of the study and give reasons why participants did not attend a wave. Only three papers [30,34,35] followed the STROBE guidelines fully. The majority of papers did not provide a reason or comment for why study participants did not attend each wave of follow-up. Sterne et al. [12] recommend that the reasons for missing data be described with respect to other variables and that authors investigate potentially important differences between participants and non-participants.

The STROBE guidelines were published in 2007. Of the nine papers published after 2007, only one followed the STROBE guidelines fully. This suggests that either journal editors are not using these guidelines or authors are not considering the impact of missing covariate data in their research.

A review of missing data in cancer prognostic studies published in 2004 by Burton et al. [36] and a review of developmental psychology studies published in 2009 by Jelicic et al. [3] reported similar findings to ours. Burton et al. [36] found a deficiency in the reporting of missing covariate data in cancer prognostic studies. After reviewing 100 articles, they found that only 40% of articles provided information about the method used to handle missing covariate data and only 12 articles would have satisfied their proposed guidelines for the reporting of missing data. We observed in our review, of articles published from 2000 to 2009, that a larger proportion of articles reported the method used to handle the missing data in the analysis but that many articles were still not reporting the amount of missing data and the reasons for missingness.

The cohort studies we identified used numerous methods to handle missing data in the exposure-outcome analyses. Although some studies used advanced statistical modelling procedures (e.g. MI and Bayesian), the majority removed individuals with missing data and performed a complete-case analysis; a method that may produce biased results if the missing data are not MCAR. Jelicic et al. also found in their review that a large proportion of studies used complete-case analysis to handle their missing data [3]. For studies with a large proportion of missing data, excluding participants with missing data may also reduce the precision of the analysis substantially. *Ad hoc* methods (e.g. LOCF, the missing indicator method and mean value substitution), which are generally not recommended [16,25] because they fail to account for the uncertainty in the data and may produce biased estimates [12], continue to be used. Although MI is becoming more accessible, only five studies used this method. The reporting of the imputation procedure was inconsistent and often incomplete. This was also observed by two independent reviews of the reporting of MI in the medical journals: *BMJ, JAMALancet* and the *New England Journal of Medicine*[12,37]. Future studies should follow the recommendations outlined by Sterne et al. [12] to ensure that enough details are provided about the MI procedure, especially the implementation and details of the imputation modelling process.

### Strengths and limitations of the literature review

We aimed to complete a comprehensive review of all papers published that analysed exposure variables measured at multiple follow-up waves. Several keywords were used in order to obtain as many articles as possible. The keyword search was then supplemented with cohort studies identified from a pooled analysis of 61 cohort studies. Although a large number of abstracts and studies were identified, some cohort studies might have been missed. If multiple papers were identified from one study, the most recent article was included in the review, which might have led us to omit papers from the same study that used a more appropriate missing data method. Our search criteria only included papers written in English and only PubMed was searched. Our search strategy was limited to articles published between 2000 and 2009. On average three papers of the type we focussed on were published each year from 2000 to 2002 and the number has increased since then, so it seems unlikely that many papers were published before this time. Also, MI was not as accessible prior to 1997, so papers published before 2000 were more likely to have used complete case analysis or other *ad hoc* methods.

## Conclusions

With the increase in the number of cohort studies analysing data with multiple follow-up waves it is essential that authors follow the STROBE guidelines [10,11] in conjunction with the guidelines proposed by Sterne et al. [12] to report on the amount of missing data in the study and the methods used to handle the missing data in the analyses. This will ensure that missing data are reported with enough detail to allow readers to assess the validity of the results. Incomplete data and the statistical methods used to deal with the missing data can lead to bias, or be inefficient, and so authors should be encouraged to use online supplements (if necessary) as a way of publishing both the details of the missing data in their study and the details of the methods used to deal with the missing data.

## Competing interests

The authors declare that they have no competing interests.

## Authors’ contributions

AK drafted the protocol for the review, reviewed the articles and drafted the manuscript. JAS conceived of the review, resolved any discrepancies encountered by AK when reviewing the articles and helped with drafting the manuscript. LB, JBC and DRE provided feedback on the design of the protocol and drafts of the manuscript. All authors read and approved the final manuscript.

## Supplementary Material

Additional file 1 **Table S1.** Detailed characteristics of the studies included in the systematic review. Details of studies included in the systematic review and the corresponding reference list [29-35,38-112]. Click here for file
