# Supplementary material for: A review of the reporting and handling of missing data in cohort studies with repeated assessment of exposure measures
Source: BMC Med Res Methodol. 2012 Jul 11;12:96. doi: 10.1186/1471-2288-12-96 (PMC3464662; doi:10.1186/1471-2288-12-96)
Supplement: Additional file 1 — Table S1. Detailed characteristics of the studies included in the systematic review. Details of studies included in the systematic review and the corresponding reference list [29-35,38-112]. [file 1471-2288-12-96-S1.doc]

Additional table 1: Detailed characteristics of the studies included in the systematic review

| **Author (year)** | **Size of baseline** | **Date of baseline recruitment** | **Number of follow-up waves used in the analysis** | **Main statistical analysis for epidemiological question†** | **Was the number of participants lost to follow-up reported?** | **Was a comment included about why the data were missing at follow-up?** | **Did the authors compare the baseline covariates for those with and without missing data?** | **What method was used to deal with the missing data at follow up waves?** | **Was a sensitivity analysis performed (for the missing data)?** |
| --- | --- | --- | --- | --- | --- | --- | --- | --- | --- |
| Agrawal (2006) [38] | Sample size not given | 1995 | 1 | Discrete- time survival analysis using proportional odds model - time-varying covariates | No | No | No | Complete case analysis | No |
| Anstey (2003) [39] | 2,087 | 1992-1993 | 3 | Structural equation modeling | Yes | No | No | Likelihood based method using all available outcome data | Yes |
| Arifeen (2001) [40] | 1,680 | November 1993 - June 1995 | 5 | Cox proportional hazards regression – time-varying covariates | Yes | No | No | Complete case analysis | No |
| Bada (2007) [41] | 8,627 | 1993-1995 | 3 | Linear mixed effects model - includes repeated assessments of covariates | Yes | No | Yes | Likelihood based method using all available outcome data | No |
| Beesdo (2007) [42] | 3,021 | 1995 | 3 | Cox proportional hazards regression – time-varying covariates | Yes | Yes | No | Complete case analysis | No |
| Berecki-Gisolf (2009) [43] | 13,716 | 1996 | 4 | GEE - logistic regression model includes repeated assessments of covariates | Yes | Yes (gave one reason for missing data) | Yes (compared for those with and without missing menopause status) | GEE - all available outcome data | No |
| Blazer (2007) [44] | 4,162 | 1986/1987 | 3 | Linear mixed effects model - includes repeated assessments of covariates | Yes | No | Yes (compared the completers to the full set at baseline) | Likelihood based method using all available outcome data | No |
| Bond (2005) [29] | 1,985 | May 1992-September 1994 | 4 | Linear mixed effects model - includes repeated assessments of covariates | Yes | No | Yes | Multiple Imputation | No |
| Bray (2000) [45] | 7,550 | 1985-1986 and 1986-1987 | 3 | Separate logistic regression models for each repeated assessment of covariates | Yes | Yes | No | Complete case analysis | No |
| Breslau (2005) [46] | 1,007 | 1989 | 3 | GEE - logistic regression model includes repeated assessments of covariates | Yes | No | Yes (brief statement in text only) | GEE - all available outcome data | No |
| Brown (2002) [47] | 2,200 | November 1987 | 3 | Logistic mixed effects model - includes repeated assessments of covariates | Yes | Yes | No | Likelihood based method using all available outcome data | No |
| Bruckl (2007) [48] | 3,021 | 1994 | 2 | Cox proportional hazards regression – time-varying covariates | Yes | No | No | Complete case analysis | No |
| Cauley (2009) [49] | 9,704 | September 1986 to October 1988 | 4 | Linear mixed effects model - includes repeated assessments of covariates | Yes | No | No | Mean value substitution | Yes |
| Celentano (2001) [50] | 2,921 | Feb 1988 and March 1989 | 11 | GEE - logistic regression models includes repeated assessments of covariates | No | No | No | Complete case analysis | No |
| Chao (2009) [51] | 5,622 | 1984-1985;  1987-1991; and 2001-2003. | multiple | Cox proportional hazards regression – time-varying covariate | No | No | No | Complete case analysis (assumed) | No |
| Cheung (2002) [52] | 17,000 | March 1958 | 6 | GEE – logistic and linear regression models includes repeated assessment of covariates | Yes | No | No | GEE – all available outcome data | No |
| Chien (2005) [53] | 3,602 | 1990 | 2 | Cox proportional hazards regression – time-varying covariates | Yes | No | No | Complete case analysis (assumed) | No |
| Clays (2007) [54] | 2,821 | 1995-1998 | 1 | Standard logistic regression - single measure of covariate derived from repeated assessments | Yes | No | No | Complete case analysis | No |
| Conron (2009) [55] | 3,532 families | 1999 or 2000 | 2 | Linear mixed effects model - includes repeated assessments of covariates | Yes | No | Yes (provided table with characteristics at each wave) | Complete case analysis | No |
| Cuddy (2006) [56] | 3,983 | 1948 | Multiple | Cox proportional hazards regression – time-varying covariates | No | No | No | Not stated | No |
| Daniels (2004) [57] | 3,080 | May 1^st^, 1983 to April 30^th^, 1984 | 16 | Standard logistic regression - single measure of covariate derived from repeated assessments | Yes | Yes | Yes | Complete case analysis | No |
| de Mutsert (2009) [58] | 1,819 | 1997 | Multiple | Cox proportional hazards regression – time-varying covariates | No | No | No | Last value carried forward | No |
| de Stavola (2007) [59] | 3,108 | 1972 to 1978 | 1 | Cox proportional hazards regression- single measure of covariate derived from repeated assessments | Yes | No | Yes (data not shown) | Complete case analysis | No |
| di Nisio (2006) [60] | 1,638 | 2008 (not stated in the paper) | 5 | Cox proportional hazards regression – time-varying covariates | No | No | No | Last value carried forward | No |
| Engberg (2006) [61] | 1,208 | 1995-1997 (not stated in the paper) | 4 | Logistic mixed effects model - includes repeated assessments of covariates | Yes | No | No | Likelihood based method using all available outcome data | No |
| Fergusson (2008) [62] | 1,265 | Mid-1977 | 20 | GEE – logistic regression models includes repeated assessments of covariates | Yes | No | Yes (data not shown) | Complete case analysis | Yes |
| Fuhrer (2003) [34] | 3,777 | 1988 | 3 | GEE – logistic regression models includes repeated assessments of covariates | Yes | No | Yes | Inverse weighted probability method for probability of remaining in study at follow-up visit | Yes |
| Fung (2009) [63] | 121,700 | 1976 (1980 used as the baseline for this analysis) | 6 | Cox proportional hazards regression – time-varying covariates | No | No | No | Complete case analysis | No |
| Gallo (2006) [64] | 12,521 | 1992 | 3 | Linear regression (method not specified for dealing with multilevel data) | Yes | No | Yes (compared completers with baseline sample) | Not stated | No |
| Gauderman (2004) [65] | 1,759 | 1993 | 4 | Linear mixed effects models includes repeated assessments of covariates | Yes | Yes (gave one reason for missing data) | Yes (web appendix) | Likelihood based method using all available outcome data | No |
| Glotzer (2009) [66] | 2,813 | November 2003 - June 2007 | Multiple | Cox proportional hazards regression – time-varying covariates | Yes | No | No | Complete case analysis | No |
| Gunderson (2009) [67] | 5,115 | 1985/6 | 4 | Generalized linear model for binary outcome with a complementary log-log link function applied to interval-censored outcome data – time-varying covariates | Yes | No | No | Not stated | No |
| Haag (2008) [68] | 7,983 | 1990-1993 | 3 | Cox proportional hazards regression – time-varying covariates | No | No | No | Complete case analysis (assumed) | No |
| Hart (2001) [69] | Renfrew/Paisley: 15,406 Collaborative study: 5,766 | Renfrew/Paisley: 1972-1976; Collaborative study: 1970-1973 | 1 | Cox proportional hazards regression - single measure of covariate derived from repeated assessments | Yes | No | No | Complete case analysis | No |
| Hogg (2006) [70] | 1,312 | August 1996 – September 1999 | Multiple | Cox proportional hazards regression – time-varying covariates | No | No | No | Complete case analysis (assumed) | No |
| Jacobs (2005) [71] | 97,786 | 1992-1993 | 3 | Cox proportional hazards regression – time-varying covariates | Yes | No | No | Complete case analysis | No |
| Jamrozik (2009) [72] | 3,284 | 1981 | 1 | Standard logistic regression – single measure of covariate derived from repeated assessments | Yes | No | No | Complete case analysis | No |
| Jimenez (2009) [73] | 1,231 | 1969-1973 | Multiple | Cox proportional hazards regression – time-varying covariates | No | No | No | Last value carried forward for exposures updated every 3 years and missing indicator methods for subjects where a covariate from a recent previous exam was not available | No |
| Juhaeri (2002) [74] | 15,792 | 1987-1990 | 3 | Cox proportional hazards – time varying covariates; and Linear mixed effects model - includes repeated assessments of covariates (NB – two outcomes assessed) | Yes | No | No | Complete case analysis | No |
| Karlamangla (2006) [75] | 14,407 | 1971-1975 | 3 | GEE – logistic regression models includes repeated assessments of covariates | Yes | No | No | GEE - all available outcome data | No |
| Keller (2007) [76] | 4,856 | Female-female twin pairs: 1988-89; Male-male and Male-female twin pairs: 1993-96 | Female-female twin pairs: up to 4 interviews; Male-male and Male-female twin pairs: up to 2 interviews | Multivariate Analysis of Variance | Yes | Yes | No | Mean value substitution; EM algorithm for symptom data | Yes |
| Kersting (2001) [77] | Sample size not given. | 1984 | 3 | Cox proportional hazards regression – time-varying covariates | No | No | No | Not stated | No |
| Kivimaki (2009) [30] | 10,308 | 1985-1988 | 3 | Standard logistic regression - single measure of covariate derived from repeated assessments | Yes | Yes | Yes (not for each wave of follow-up) | Complete case analysis (Multiple Imputation used for sensitivity analysis) | Yes |
| Lacson (2009) [78] | 79,545 | 2007 | 12 | Cox proportional hazards regression – time-varying covariates | No | No | No | Complete case analysis | No |
| Lamarca (2003) [79] | 1,315 | 1986-1987 | 1 | Cox proportional hazards regression – time-varying covariates | Yes | No | No | Last value carried forward | No |
| Lawson (2008) [80] | 14,062 | April 1991 to December 1992 | Multiple | Linear mixed effects model - includes repeated assessments of covariates | Yes | No | No | Mean value substitution | No |
| Lee (2006) [81] | 11,234 | 1996 | 7 | Linear mixed effects model - includes repeated assessments of covariates | Yes | No | No | Complete case analysis | Yes |
| Lee (2007) [82] | 5,124 | 1971 | 1 | Cox proportional hazards regression – time-varying covariates | Yes | No | No | Complete case analysis | No |
| Li (2004) [83] | 2,581 | 1994-1996 | Multiple | Cox proportional hazards regression – time-varying covariates | Yes | No | Yes (compared the completers to the full set at baseline) | Complete case analysis (assumed) | No |
| Li (2009) [84] | 4,856 | October 1998-September 2003 | at least 3 | Linear mixed effects model - includes repeated assessments of covariates | Yes | No | No | Last value carried forward | No |
| Limburg (2005) [85] | 41,836 | 1986 | 4 | Cox proportional hazards regression – time-varying covariates | Yes | No | No | Complete case analysis (assumed) | No |
| Luchenski (2008) [86] | 17,276 | 1994-1995 | 4 | GEE –linear regression model includes repeated assessments of covariates | Yes | No | No | GEE - all available outcome data | Yes |
| McCormack (2003) [31] | 2,547 | 3-9 March 1946 | 10 | Logistic mixed effects model - includes repeated assessments of covariates | Yes | Yes | No | Multiple Imputation | No |
| Melamed (2006) [87] | 1,041 | October 1995-June 1998 | 4 | Cox proportional hazards regression –time-varying covariates | No | No | No | Last value carried forward | Yes |
| Menotti (2008) [88] | 12,763 | 1957-1964 | 5 | Cox proportional hazards regression – time varying covariates and single measure of covariate derived from repeated assessments | Yes | No | No | Not stated | No |
| Michaelsson (2007) [89] | 2,322 | 1970-1973 | 4 | Cox proportional hazards regression – time varying covariates | Yes | No | No | Complete case analysis (assumed) | Yes |
| Michaud (2008) [90] | 51,529 | 1986 | Multiple | Cox proportional hazards regression – time-varying covariates | Yes | No | No | Complete case analysis (assumed) | No |
| Mirzaei (2007) [91] | 1,230 | 1994 | 1 | Standard linear regression - single measure of covariate derived from repeated assessments | Yes | No | Yes (data not shown) | Complete case analysis | No |
| Mishra (2006) [92] | 5,362 | March 1946 | 3 | Linear mixed effects models includes repeated assessments of covariates | No | No | Yes (data not shown) | Likelihood based method using all available outcome data | No |
| Monda (2008) [93] | 7,405 | 1989 but 1991 used as baseline in this analysis | 3 | Linear mixed effects models includes repeated assessments of covariates | Yes | No | No | Likelihood based method using all available outcome data | No |
| Moss (2008) [94] | 4,926 | March 1988 – September 1989; 1993 used as baseline for this study | 2 | Standard logistic regression - single measure of covariate derived from repeated assessments | Yes | Yes | Yes (in previous paper) | Complete case analysis | No |
| Nabi (2005) [95] | 20,626 | 1989 | Multiple | Cox proportional hazards regression – time-varying covariates | Yes | No | No | Complete case analysis | No |
| Nakano (2005) [96] | 2,987 | April 1985-March 1990 | 10 | Repeated- measures Analysis of Variance | Yes | No | No | Complete case analysis | No |
| Nowicki (2008) [97] | 2,628 (2,059 included in this anlaysis) | 1994-1995 | 1 to 7 | Cox proportional hazards regression – time-varying covariates | No | No | No | Complete case analysis (assumed) | No |
| Ormel (2004) [98] | 7,076 | 1996 | 2 | Repeated-measures Analysis of Variance | Yes | No | Yes (data not shown) | Complete case analysis | No |
| Rabbitt (2008) [99] | 2,620 | 1983 – 1984 | Multiple | Andersen-Gill model (Cox) – time- varying covariates | Yes | No | No | Complete case analysis | No |
| Randolph (2005) [100] | 3,302 | 1996-1997 (not stated in the paper) | 4 | GEE – logistic regression models includes repeated assessments of covariates | Yes | No | Yes (compared certain characteristics for participants at each wave) | GEE - all available outcome data | No |
| Rousseau (2003) [101] | 2,528 | 1993-1997 | Multiple | GEE – logistic regression models includes repeated assessments of covariates | Yes | No | No | Last value carried forward | No |
| Ryu (2009) [102] | 15,347 | 2002 | 3 to 6 | Cox proportional hazards regression – time-varying covariates | Yes | No | Yes | Complete case analysis | No |
| Seid (2006) [103] | 10,241 | 2001 | 2 | GEE – linear regression models includes repeated assessments of covariates | Yes | No | Yes (compared certain characteristics for participants at each wave) | GEE - all available outcome data | No |
| Silfverdal (2007) [104] | 17,000 | 1970 | 2 | Cox proportional hazards regression – time-varying covariate | Yes | Yes (not extensive) | Yes (brief statement) | Complete case analysis | No |
| Sogaard (2007) [35] | 16,209 | 1972-1973 | 1 | Standard logistic regression - single measure of covariate derived from repeated assessments | Yes | Yes | Yes (but data not shown) | Complete case analysis | No |
| Spence (2002) [105] | 8,556 | 1981 (not stated in the paper) | 4 | Standard logistic regression - single measure of covariate derived from repeated assessments | Yes | No | Yes | Complete case analysis | No |
| Stewart (2009) [106] | Sample size not given | 1965-1968 | 6 | Linear mixed effects models includes repeated assessments of covariates | Yes | No | Yes | Complete case analysis | No |
| Strasak (2008) [107] | 184,774 | 1985-2005 | 1 to 19 | Cox proportional hazards regression – time-varying covariates | Yes | No | Yes (compared analytic sample with baseline sample) | Complete case analysis and likelihood based method using all available outcome data | No |
| Strawbridge (2000) [108] | 5,894 (this analysis) | 1965 | 3 | Cox proportional hazards regression – time-varying covariates | Yes | No | No | Complete case analysis | No |
| Sugihara (2008) [32] | 6,000; 3,973 participated in baseline for this study | 1999/2000 | 2 | GEE –linear regression models includes repeated assessments of covariates | Yes | No | No | Multiple Imputation used for some of the study variables | No |
| Sung (2004) [109] | 1,420 | (not stated in the paper) | 4 to 10 | Hierarchical Bayesian Approach | Yes | Yes | No | Included a probability function for the missing outcome variables within the Bayesian framework. | No |
| Tehard (2004) [110] | 98,997 | 1990/1991 | 5 | Cox proportional hazards regression – time-varying covariates | Yes | No | No | Complete case analysis | No |
| Vikan (2009) [111] | 6,889 - not reported in this paper | 1994-1995 (used as baseline for this study) | 1 | Standard linear regression – single measure of covariate derived from repeated assessments | No | No | No | Complete case analysis | No |
| Wang (2008) [112] | 1,337 | 1948-1964 | up to 12 | Cox proportional hazards regression – time-varying covariates | Yes | No | No | Complete case analysis | No |
| Wiles (2007) [33] | 2,398 | Phase II: 1984-1988 (used as the baseline for this study) | 2 | Standard logistic regression – single measure of covariate derived from repeated assessments | Yes | No | No | Complete case analysis (Multiple Imputation used for sensitivity analysis) | Yes |

GEE – Generalised Estimating Equations;

† The description of the statistical analysis used for the epidemiological question is based on our interpretation of the information provided in the methods section of each paper.

**References**

29. Bond GE, Burr RL, McCurry SM, Rice MM, Borenstein AR, Larson EB: **Alcohol and cognitive performance: a longitudinal study of older Japanese Americans. The Kame Project.** *Int Psychogeriatr* 2005, **17:**653-668.

30. Kivimaki M, Lawlor DA, Singh-Manoux A, Batty GD, Ferrie JE, Shipley MJ, Nabi H, Sabia S, Marmot MG, Jokela M: **Common mental disorder and obesity: insight from four repeat measures over 19 years: prospective Whitehall II cohort study.** *BMJ* 2009, **339:**b3765.

31. McCormack VA, dos Santos Silva I, De Stavola BL, Perry N, Vinnicombe S, Swerdlow AJ, Hardy R, Kuh D: **Life-course body size and perimenopausal mammographic parenchymal patterns in the MRC 1946 British birth cohort.** *Br J Cancer* 2003, **89:**852-859.

32. Sugihara Y, Sugisawa H, Shibata H, Harada K: **Productive roles, gender, and depressive symptoms: evidence from a national longitudinal study of late-middle-aged Japanese.** *J Gerontol B Psychol Sci Soc Sci* 2008, **63:**P227-P234.

33. Wiles NJ, Haase AM, Gallacher J, Lawlor DA, Lewis G: **Physical activity and common mental disorder: results from the Caerphilly study.** *Am J Epidemiol* 2007, **165:**946-954.

34. Fuhrer R, Dufouil C, Dartigues JF: **Exploring sex differences in the relationship between depressive symptoms and dementia incidence: prospective results from the PAQUID Study.** *J Am Geriatr Soc* 2003, **51:**1055-1063.

35. Sogaard AJ, Meyer HE, Tonstad S, Haheim LL, Holme I: **Weight cycling and risk of forearm fractures: a 28-year follow-up of men in the Oslo Study.** *Am J Epidemiol* 2008, **167:**1005-1013.

38. Agrawal A, Grant JD, Waldron M, Duncan AE, Scherrer JF, Lynskey MT, Madden PA, Bucholz KK, Heath AC: **Risk for initiation of substance use as a function of age of onset of cigarette, alcohol and cannabis use: findings in a Midwestern female twin cohort.** *Prev Med* 2006, **43:**125-128.

39. Anstey KJ, Hofer SM, Luszcz MA: **Cross-sectional and longitudinal patterns of dedifferentiation in late-life cognitive and sensory function: the effects of age, ability, attrition, and occasion of measurement.** *J Exp Psychol Gen* 2003, **132:**470-487.

40. Arifeen S, Black RE, Antelman G, Baqui A, Caulfield L, Becker S: **Exclusive breastfeeding reduces acute respiratory infection and diarrhea deaths among infants in Dhaka slums.** *Pediatrics* 2001, **108:**E67.

41. Bada HS, Das A, Bauer CR, Shankaran S, Lester B, LaGasse L, Hammond J, Wright LL, Higgins R: **Impact of prenatal cocaine exposure on child behavior problems through school age.** *Pediatrics* 2007, **119:**e348-359.

42. Beesdo K, Bittner A, Pine DS, Stein MB, Hofler M, Lieb R, Wittchen HU: **Incidence of social anxiety disorder and the consistent risk for secondary depression in the first three decades of life.** *Arch Gen Psychiatry* 2007, **64:**903-912.

43. Berecki-Gisolf J, Begum N, Dobson AJ: **Symptoms reported by women in midlife: menopausal transition or aging?** *Menopause* 2009, **16:**1021-1029.

44. Blazer DG, Sachs-Ericsson N, Hybels CF: **Perception of unmet basic needs as a predictor of depressive symptoms among community-dwelling older adults.** *J Gerontol A Biol Sci Med Sci* 2007, **62:**191-195.

45. Bray JW, Zarkin GA, Ringwalt C, Qi J: **The relationship between marijuana initiation and dropping out of high school.** *Health Econ* 2000, **9:**9-18.

46. Breslau N, Schultz LR, Johnson EO, Peterson EL, Davis GC: **Smoking and the risk of suicidal behavior: a prospective study of a community sample.** *Arch Gen Psychiatry* 2005, **62:**328-334.

47. Brown JW, Liang J, Krause N, Akiyama H, Sugisawa H, Fukaya T: **Transitions in living arrangements among elders in Japan: does health make a difference?** *J Gerontol B Psychol Sci Soc Sci* 2002, **57:**S209-220.

48. Bruckl TM, Wittchen HU, Hofler M, Pfister H, Schneider S, Lieb R: **Childhood separation anxiety and the risk of subsequent psychopathology: Results from a community study.** *Psychother Psychosom* 2007, **76:**47-56.

49. Cauley JA, Lui LY, Barnes D, Ensrud KE, Zmuda JM, Hillier TA, Hochberg MC, Schwartz AV, Yaffe K, Cummings SR, Newman AB: **Successful skeletal aging: a marker of low fracture risk and longevity. The Study of Osteoporotic Fractures (SOF).** *J Bone Miner Res* 2009, **24:**134-143.

50. Celentano DD, Munoz A, Cohn S, Vlahov D: **Dynamics of behavioral risk factors for HIV/AIDS: a 6-year prospective study of injection drug users.** *Drug Alcohol Depend* 2001, **61:**315-322.

51. Chao C, Jacobson LP, Tashkin D, Martinez-Maza O, Roth MD, Margolick JB, Chmiel JS, Holloway MN, Zhang ZF, Detels R: **Recreational amphetamine use and risk of HIV-related non-Hodgkin lymphoma.** *Cancer Causes Control* 2009, **20:**509-516.

52. Cheung YB, Khoo KS, Karlberg J, Machin D: **Association between psychological symptoms in adults and growth in early life: longitudinal follow up study.** *BMJ* 2002, **325:**749.

53. Chien KL, Hsu HC, Sung FC, Su TC, Chen MF, Lee YT: **Hyperuricemia as a risk factor on cardiovascular events in Taiwan: The Chin-Shan Community Cardiovascular Cohort Study.** *Atherosclerosis* 2005, **183:**147-155.

54. Clays E, De Bacquer D, Leynen F, Kornitzer M, Kittel F, De Backer G: **Job stress and depression symptoms in middle-aged workers--prospective results from the Belstress study.** *Scand J Work Environ Health* 2007, **33:**252-259.

55. Conron KJ, Beardslee W, Koenen KC, Buka SL, Gortmaker SL: **A longitudinal study of maternal depression and child maltreatment in a national sample of families investigated by child protective services.** *Arch Pediatr Adolesc Med* 2009, **163:**922-930.

56. Cuddy TE, Tate RB: **Sudden unexpected cardiac death as a function of time since the detection of electrocardiographic and clinical risk factors in apparently healthy men: the Manitoba Follow-Up Study, 1948 to 2004.** *Can J Cardiol* 2006, **22:**205-211.

57. Daniels MC, Adair LS: **Growth in young Filipino children predicts schooling trajectories through high school.** *J Nutr* 2004, **134:**1439-1446.

58. de Mutsert R, Grootendorst DC, Boeschoten EW, Brandts H, van Manen JG, Krediet RT, Dekker FW: **Subjective global assessment of nutritional status is strongly associated with mortality in chronic dialysis patients.** *Am J Clin Nutr* 2009, **89:**787-793.

59. De Stavola BL, Meade TW: **Long-term effects of hemostatic variables on fatal coronary heart disease: 30-year results from the first prospective Northwick Park Heart Study (NPHS-I).** *J Thromb Haemost* 2007, **5:**461-471.

60. Di Nisio M, Barbui T, Di Gennaro L, Borrelli G, Finazzi G, Landolfi R, Leone G, Marfisi R, Porreca E, Ruggeri M, et al: **The haematocrit and platelet target in polycythemia vera.** *Br J Haematol* 2007, **136:**249-259.

61. Engberg J, Morral AR: **Reducing substance use improves adolescents' school attendance.** *Addiction* 2006, **101:**1741-1751.

62. Fergusson DM, Boden JM, Horwood LJ: **The developmental antecedents of illicit drug use: evidence from a 25-year longitudinal study.** *Drug Alcohol Depend* 2008, **96:**165-177.

63. Fung TT, Malik V, Rexrode KM, Manson JE, Willett WC, Hu FB: **Sweetened beverage consumption and risk of coronary heart disease in women.** *Am J Clin Nutr* 2009, **89:**1037-1042.

64. Gallo WT, Bradley EH, Dubin JA, Jones RN, Falba TA, Teng HM, Kasl SV: **The persistence of depressive symptoms in older workers who experience involuntary job loss: results from the health and retirement survey.** *J Gerontol B Psychol Sci Soc Sci* 2006, **61:**S221-228.

65. Gauderman WJ, Avol E, Gilliland F, Vora H, Thomas D, Berhane K, McConnell R, Kuenzli N, Lurmann F, Rappaport E, et al: **The effect of air pollution on lung development from 10 to 18 years of age.** *N Engl J Med* 2004, **351:**1057-1067.

66. Glotzer TV, Daoud EG, Wyse DG, Singer DE, Ezekowitz MD, Hilker C, Miller C, Qi D, Ziegler PD: **The relationship between daily atrial tachyarrhythmia burden from implantable device diagnostics and stroke risk: the TRENDS study.** *Circ Arrhythm Electrophysiol* 2009, **2:**474-480.

67. Gunderson EP, Jacobs DR, Jr., Chiang V, Lewis CE, Tsai A, Quesenberry CP, Jr., Sidney S: **Childbearing is associated with higher incidence of the metabolic syndrome among women of reproductive age controlling for measurements before pregnancy: the CARDIA study.** *Am J Obstet Gynecol* 2009, **201:**177 e171-179.

68. Haag MD, Bos MJ, Hofman A, Koudstaal PJ, Breteler MM, Stricker BH: **Cyclooxygenase selectivity of nonsteroidal anti-inflammatory drugs and risk of stroke.** *Arch Intern Med* 2008, **168:**1219-1224.

69. Hart CL, Hole DJ, Davey Smith G: **Are two really better than one? Empirical examination of repeat blood pressure measurements and stroke risk in the Renfrew/Paisley and collaborative studies.** *Stroke* 2001, **32:**2697-2699.

70. Hogg RS, Bangsberg DR, Lima VD, Alexander C, Bonner S, Yip B, Wood E, Dong WW, Montaner JS, Harrigan PR: **Emergence of drug resistance is associated with an increased risk of death among patients first starting HAART.** *PLoS Med* 2006, **3:**e356.

71. Jacobs EJ, Thun MJ, Connell CJ, Rodriguez C, Henley SJ, Feigelson HS, Patel AV, Flanders WD, Calle EE: **Aspirin and other nonsteroidal anti-inflammatory drugs and breast cancer incidence in a large U.S. cohort.** *Cancer Epidemiol Biomarkers Prev* 2005, **14:**261-264.

72. Jamrozik E, Knuiman MW, James A, Divitini M, Musk AW: **Risk factors for adult-onset asthma: a 14-year longitudinal study.** *Respirology* 2009, **14:**814-821.

73. Jimenez M, Krall EA, Garcia RI, Vokonas PS, Dietrich T: **Periodontitis and incidence of cerebrovascular disease in men.** *Ann Neurol* 2009, **66:**505-512.

74. Juhaeri, Stevens J, Chambless LE, Nieto FJ, Jones D, Schreiner P, Arnett D, Cai J: **Associations of weight loss and changes in fat distribution with the remission of hypertension in a bi-ethnic cohort: the Atherosclerosis Risk in Communities Study.** *Prev Med* 2003, **36:**330-339.

75. Karlamangla A, Zhou K, Reuben D, Greendale G, Moore A: **Longitudinal trajectories of heavy drinking in adults in the United States of America.** *Addiction* 2006, **101:**91-99.

76. Keller MC, Neale MC, Kendler KS: **Association of different adverse life events with distinct patterns of depressive symptoms.** *Am J Psychiatry* 2007, **164:**1521-1529; quiz 1622.

77. Kersting RC: **Impact of social support, diversity, and poverty on nursing home utilization in a nationally representative sample of older Americans.** *Soc Work Health Care* 2001, **33:**67-87.

78. Lacson E, Jr., Wang W, Lazarus JM, Hakim RM: **Change in vascular access and mortality in maintenance hemodialysis patients.** *Am J Kidney Dis* 2009, **54:**912-921.

79. Lamarca R, Ferrer M, Andersen PK, Liestol K, Keiding N, Alonso J: **A changing relationship between disability and survival in the elderly population: differences by age.** *J Clin Epidemiol* 2003, **56:**1192-1201.

80. Lawson DW, Mace R: **Sibling configuration and childhood growth in contemporary British families.** *Int J Epidemiol* 2008, **37:**1408-1421.

81. Lee DH, Ha MH, Kam S, Chun B, Lee J, Song K, Boo Y, Steffen L, Jacobs DR, Jr.: **A strong secular trend in serum gamma-glutamyltransferase from 1996 to 2003 among South Korean men.** *Am J Epidemiol* 2006, **163:**57-65.

82. Lee DS, Evans JC, Robins SJ, Wilson PW, Albano I, Fox CS, Wang TJ, Benjamin EJ, D'Agostino RB, Vasan RS: **Gamma glutamyl transferase and metabolic syndrome, cardiovascular disease, and mortality risk: the Framingham Heart Study.** *Arterioscler Thromb Vasc Biol* 2007, **27:**127-133.

83. Li G, Higdon R, Kukull WA, Peskind E, Van Valen Moore K, Tsuang D, van Belle G, McCormick W, Bowen JD, Teri L, et al: **Statin therapy and risk of dementia in the elderly: a community-based prospective cohort study.** *Neurology* 2004, **63:**1624-1628.

84. Li LW, Conwell Y: **Effects of changes in depressive symptoms and cognitive functioning on physical disability in home care elders.** *J Gerontol A Biol Sci Med Sci* 2009, **64:**230-236.

85. Limburg PJ, Anderson KE, Johnson TW, Jacobs DR, Jr., Lazovich D, Hong CP, Nicodemus KK, Folsom AR: **Diabetes mellitus and subsite-specific colorectal cancer risks in the Iowa Women's Health Study.** *Cancer Epidemiol Biomarkers Prev* 2005, **14:**133-137.

86. Luchenski S, Quesnel-Vallee A, Lynch J: **Differences between women's and men's socioeconomic inequalities in health: longitudinal analysis of the Canadian population, 1994-2003.** *J Epidemiol Community Health* 2008, **62:**1036-1044.

87. Melamed ML, Eustace JA, Plantinga L, Jaar BG, Fink NE, Coresh J, Klag MJ, Powe NR: **Changes in serum calcium, phosphate, and PTH and the risk of death in incident dialysis patients: a longitudinal study.** *Kidney Int* 2006, **70:**351-357.

88. Menotti A, Lanti M, Kromhout D, Blackburn H, Jacobs D, Nissinen A, Dontas A, Kafatos A, Nedeljkovic S, Adachi H: **Homogeneity in the relationship of serum cholesterol to coronary deaths across different cultures: 40-year follow-up of the Seven Countries Study.** *Eur J Cardiovasc Prev Rehabil* 2008, **15:**719-725.

89. Michaelsson K, Olofsson H, Jensevik K, Larsson S, Mallmin H, Berglund L, Vessby B, Melhus H: **Leisure physical activity and the risk of fracture in men.** *PLoS Med* 2007, **4:**e199.

90. Michaud DS, Liu Y, Meyer M, Giovannucci E, Joshipura K: **Periodontal disease, tooth loss, and cancer risk in male health professionals: a prospective cohort study.** *Lancet Oncol* 2008, **9:**550-558.

91. Mirzaei M, Taylor R, Morrell S, Leeder SR: **Predictors of blood pressure in a cohort of school-aged children.** *Eur J Cardiovasc Prev Rehabil* 2007, **14:**624-629.

92. Mishra GD, McNaughton SA, Bramwell GD, Wadsworth ME: **Longitudinal changes in dietary patterns during adult life.** *Br J Nutr* 2006, **96:**735-744.

93. Monda KL, Adair LS, Zhai F, Popkin BM: **Longitudinal relationships between occupational and domestic physical activity patterns and body weight in China.** *Eur J Clin Nutr* 2008, **62:**1318-1325.

94. Moss SE, Klein R, Klein BE: **Long-term incidence of dry eye in an older population.** *Optom Vis Sci* 2008, **85:**668-674.

95. Nabi H, Consoli SM, Chastang JF, Chiron M, Lafont S, Lagarde E: **Type A behavior pattern, risky driving behaviors, and serious road traffic accidents: a prospective study of the GAZEL cohort.** *Am J Epidemiol* 2005, **161:**864-870.

96. Nakano T, Tatemichi M, Miura Y, Sugita M, Kitahara K: **Long-term physiologic changes of intraocular pressure: a 10-year longitudinal analysis in young and middle-aged Japanese men.** *Ophthalmology* 2005, **112:**609-616.

97. Nowicki MJ, Vigen C, Mack WJ, Seaberg E, Landay A, Anastos K, Young M, Minkoff H, Greenblatt R, Levine AM: **Association of cells with natural killer (NK) and NKT immunophenotype with incident cancers in HIV-infected women.** *AIDS Res Hum Retroviruses* 2008, **24:**163-168.

98. Ormel J, Oldehinkel AJ, Vollebergh W: **Vulnerability before, during, and after a major depressive episode: a 3-wave population-based study.** *Arch Gen Psychiatry* 2004, **61:**990-996.

99. Rabbitt P, Lunn M, Wong D, Cobain M: **Sudden declines in intelligence in old age predict death and dropout from longitudinal studies.** *J Gerontol B Psychol Sci Soc Sci* 2008, **63:**P205-P211.

100. Randolph JF, Jr., Sowers M, Bondarenko I, Gold EB, Greendale GA, Bromberger JT, Brockwell SE, Matthews KA: **The relationship of longitudinal change in reproductive hormones and vasomotor symptoms during the menopausal transition.** *J Clin Endocrinol Metab* 2005, **90:**6106-6112.

101. Rousseau MC, Abrahamowicz M, Villa LL, Costa MC, Rohan TE, Franco EL: **Predictors of cervical coinfection with multiple human papillomavirus types.** *Cancer Epidemiol Biomarkers Prev* 2003, **12:**1029-1037.

102. Ryu S, Chang Y, Woo HY, Lee KB, Kim SG, Kim DI, Kim WS, Suh BS, Jeong C, Yoon K: **Time-dependent association between metabolic syndrome and risk of CKD in Korean men without hypertension or diabetes.** *Am J Kidney Dis* 2009, **53:**59-69.

103. Seid M, Varni JW, Cummings L, Schonlau M: **The impact of realized access to care on health-related quality of life: a two-year prospective cohort study of children in the California State Children's Health Insurance Program.** *J Pediatr* 2006, **149:**354-361.

104. Silfverdal SA, Ehlin A, Montgomery SM: **Protection against clinical pertussis induced by whole-cell pertussis vaccination is related to primo-immunisation intervals.** *Vaccine* 2007, **25:**7510-7515.

105. Spence SH, Najman JM, Bor W, O'Callaghan MJ, Williams GM: **Maternal anxiety and depression, poverty and marital relationship factors during early childhood as predictors of anxiety and depressive symptoms in adolescence.** *J Child Psychol Psychiatry* 2002, **43:**457-469.

106. Stewart R, Xue QL, Masaki K, Petrovitch H, Ross GW, White LR, Launer LJ: **Change in blood pressure and incident dementia: a 32-year prospective study.** *Hypertension* 2009, **54:**233-240.

107. Strasak AM, Kelleher CC, Klenk J, Brant LJ, Ruttmann E, Rapp K, Concin H, Diem G, Pfeiffer KP, Ulmer H: **Longitudinal change in serum gamma-glutamyltransferase and cardiovascular disease mortality: a prospective population-based study in 76,113 Austrian adults.** *Arterioscler Thromb Vasc Biol* 2008, **28:**1857-1865.

108. Strawbridge WJ, Cohen RD, Shema SJ: **Comparative strength of association between religious attendance and survival.** *Int J Psychiatry Med* 2000, **30:**299-308.

109. Sung M, Erkanli A, Angold A, Costello EJ: **Effects of age at first substance use and psychiatric comorbidity on the development of substance use disorders.** *Drug Alcohol Depend* 2004, **75:**287-299.

110. Tehard B, Lahmann PH, Riboli E, Clavel-Chapelon F: **Anthropometry, breast cancer and menopausal status: use of repeated measurements over 10 years of follow-up-results of the French E3N women's cohort study.** *Int J Cancer* 2004, **111:**264-269.

111. Vikan T, Johnsen SH, Schirmer H, Njolstad I, Svartberg J: **Endogenous testosterone and the prospective association with carotid atherosclerosis in men: the Tromso study.** *Eur J Epidemiol* 2009, **24:**289-295.

112. Wang NY, Young JH, Meoni LA, Ford DE, Erlinger TP, Klag MJ: **Blood pressure change and risk of hypertension associated with parental hypertension: the Johns Hopkins Precursors Study.** *Arch Intern Med* 2008, **168:**643-648.
